# Quantifying the neglected: Initial estimation of the global burden and economic impact of human toxocariasis

**DOI:** 10.1016/j.crpvbd.2024.100180

**Published:** 2024-05-21

**Authors:** Alistair Antonopoulos, Alessio Giannelli, Eric R. Morgan, Johannes Charlier

**Affiliations:** a Kreavet, Kruibeke, Belgium; b Queens University, Belfast, United Kingdom

**Keywords:** One Health, Epidemiology, DALY, Zoonosis, Impact, *Toxocara*

## Abstract

Toxocariasis is a parasitic zoonotic infection caused by *Toxocara* spp., ascarid nematodes of companion animals (dogs and cats) affecting people in both high-income and low/middle-income countries. Toxocariasis can manifest as several distinct syndromes. The most frequent, often termed common toxocariasis, is a self-limiting and mild febrile illness. Ocular and visceral *larva migrans* are severe disease manifestations affecting the eye and other internal organs, respectively, but their reported occurrence is rare. The vast majority of symptomatic cases are thought due to common toxocariasis, which has also been associated with cognitive impairment in children. Few studies to date have sought to quantity the health burden of toxocariasis in humans. In this study we provide a preliminary estimation using the Disability-Adjusted Life Year (DALY) approach. We estimate a total of 23,084 DALYs are lost annually in 28 selected countries due to common toxocariasis. Extrapolating based on a global average seroprevalence rate of 19%, we estimate 91,714 DALYs per year are lost across all countries due to toxocariasis, of which 40,912 are attributable to less severe forms, i.e. common toxocariasis, and 50,731 to cognitive impairment in children. Clinically diagnosed and reported ocular and visceral *larva migrans* represent a small proportion of estimated total health burden. We also found a positive correlation at national level between prevalence in cats or dogs and seroprevalence in humans, but no correlation between estimated soil contamination and seroprevalence in humans. Finally, we estimate the potential economic impact of toxocariasis in selected countries at 2.5 billion USD per annum, from costs of medical treatment and lost income. These preliminary estimates should serve as a call to action for further research and evidence-based measures to tackle toxocariasis.

## Highlights

• Toxocariasis is an important neglected zoonosis, but its burden is thought to be underestimated.
• Despite this, toxocariasis has not yet been included in Global Burden of Disease studies.
• Here, we describe the first example of using a DALY approach to estimate the global burden of toxocariasis.
• We also estimate the potential economic impact of infection due to lost earnings and medical costs.
• Toxocariasis has been associated with cognitive impairment, potentially impacting school-age children.

## Graphical abstract

**Figure:**
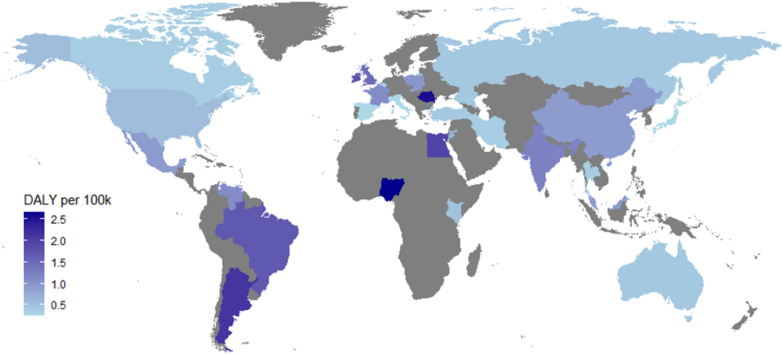

## 1 Introduction

Toxocariasis is a parasitic zoonosis caused by ascarid nematodes of dogs (*Toxocara canis*) or cats (*Toxocara cati*) (Ma et al., 2018; Hotez, 2020; Giannelli et al., 2024). Due to the broad distribution of companion animals and widespread cohabitation with humans, in addition to large feral populations of dogs and cats in sparsely inhabited regions, *Toxocara* eggs have been detected in companion animal faeces, and *Toxocara* antibodies in humans worldwide (Ma et al., 2020). Indeed, the global average (sero)prevalence in humans has been estimated at 19%, and soil contamination at 21% (Fakhri et al., 2018; Rostami et al., 2020a, 2020b; Ma et al., 2020), with the global prevalence in dogs estimated at 11% (Rostami et al., 2020a), and in stray dogs potentially as high as 50% (Daryani et al., 2009). Based on the high prevalence and known health outcomes, several authors have warned that toxocariasis is a serious and underestimated issue, potentially with impacts on human health comparable to the common soil-transmitted helminths (STHs), including hookworms, *Trichuris* and *Ascaris* (Hotez, 2020). The WHO has defined the neglected tropical diseases (NTDs) as a diverse group of 20 conditions that affect mainly people in tropical areas in impoverished communities WHO (2023). The STHs are one of those conditions that have garnered increased attention in recent decades (Hotez et al., 2008) with increased control efforts resulting in declining morbidity in several regions around the world due to the success of mass antiparasitic drug administration campaigns (Montresor et al., 2020), *Toxocara* spp. are strictly speaking not included in the STHs (Engels and Zhou, 2020), but toxocariasis is acquired in a similar way and can also be considered a disease of poverty, with socially disadvantaged populations most at risk (McGuinness and Leder, 2014). However, unlike STHs, and other neglected (tropical) diseases, toxocariasis is also prevalent in high-income countries (Hotez and Wilkins, 2009; Smith et al., 2009) and is entirely zoonotic in origin. Although it has been suggested that the burden of toxocariasis could be comparable to that of STHs (Hotez, 2020), the disease has not yet been considered in the Global Burden of Diseases Initiative (GBD, 2021), which aims to provide a comprehensive picture of both mortality and disability across the world, or in other burden assessment studies (Havelaar et al., 2015; Abdoli and Maspi, 2018; Di Bari et al., 2023). Thus, toxocariasis could be considered a doubly neglected disease, requiring more attention to better understand its true burden.

The definitive hosts for *Toxocara* spp. are mainly domestic cats and dogs for *T. cati* and *T. canis*, respectively, with adult worms residing in the intestinal lumen of the small intestine; several wild carnivore species are also definitive hosts (Holland, 2023). Transmission to humans can occur through a variety of routes, most commonly due to ingestion of infective larvated eggs in soil, sand, or water (Fakhri et al., 2018; Ma et al., 2018; Otero et al., 2018), and, in principle, also contact with and subsequent ingestion of eggs on the fur of companion animals (Öge et al., 2014), although when animals are well cared for this is much less likely (Merigueti et al., 2017). Transmission risk is consequently low (Overgaauw et al., 2009) and is mainly a risk factor in the case of stray dogs and puppies (Merigueti et al., 2017). *Toxocara* can also be ingested with food, *via* either eggs on contaminated vegetables or larvae in infected meat from animals acting as paratenic hosts (Healy et al., 2022). However, at the time of writing, there is still little known about the relative importance of different transmission routes from animals to humans, although transmission *via* soil contaminated with *Toxocara* spp. eggs after deposition of dog and cat faeces is considered to be one of the main routes of infection, particularly in children (Magnaval et al., 2001). In companion animals, which are definitive hosts, pathology generally manifests due to entero-hepatic-pulmonary migration of juvenile worms, leading to respiratory and intestinal clinical signs (Sprent, 1958), but is often mild or asymptomatic. If the parasite load is extremely heavy, animals can present with an impacted small intestine which can lead to intestinal rupture and subsequent septic peritonitis (Schnieder et al., 2011). Diarrhoea can be observed, especially when there is co-infection with one or more other pathogens (Duijvestijn et al., 2016). *Toxocara* can be a contributing cause of fading puppy syndrome (Duarte et al., 2016). As humans are not the definitive host, the parasite cannot complete its life-cycle, with infective larvae migrating through the body following hatching for months to years, leading to tissue damage within whichever tissue they happen to enter (Despommier, 2003). This can cause visceral and ocular manifestations of toxocariasis, in the latter case potentially leading to permanent vision loss (Despommier, 2003; Hotez, 2020). *Toxocara* larvae can also affect the brain and spinal cord, leading to neurotoxocariasis (NT); however, this disease manifestation is very rarely documented, with only 20 cases reported between 1950 and 2000 (Auer and Walochnik, 2020), and only 99 reported globally as of 2018 (Chen et al., 2018). Since *Toxocara* is neurotropic in rodents (Strube et al., 2020), the lack of human cases could be due to fundamental differences in host pathogenesis and/or under-reporting.

Although clinical toxocariasis in humans is rarely diagnosed (Halsby et al., 2016; Hotez, 2020), links have recently been drawn to more insidious pulmonary and neurologic disease (Hotez and Wilkins, 2009; Ma et al., 2018). Furthermore, toxocariasis can also present as a self-limiting manifestation of non-specific febrile illness accompanied by anorexia, nausea, pulmonary symptoms, and behavioural disorders, often called common toxocariasis (Ma et al., 2018; Auer and Walochnik, 2020). Finally, despite the rarity of clinical NT, associations have been drawn between toxocariasis and cognitive function in both children and adults (Walsh and Haseeb, 2012; Song et al., 2023). A particularly important study reported a relationship between *Toxocara-*specific seropositivity and performance on two different standardised cognitive test scores in children in the USA, which was independent of socioeconomic status, gender, race and rural/urban residence (Walsh and Haseeb, 2012). It is, therefore, of interest to consider the potential impact on cognitive development in school-age children as part of burden of disease estimations.

To date few studies have used the DALY approach to estimate the burden of toxocariasis. Studies that have used this approach focused on severe clinical manifestations such as visceral *larva migrans* (VLM) with laboratory confirmed diagnosis (Noguera Zayas et al., 2021). This neglects the much more frequent and arguably substantial impacts from common toxocariasis. In this study, we aimed to undertake a first comprehensive impact assessment of toxocariasis including: (i) estimation of the potential disability-adjusted life years (DALY) for all disease manifestations of toxocariasis, including the common but less severe manifestations; (ii) estimation of the potential economic cost of the disease; and (iii) exploration of the correlation between zoonotic *Toxocara* spp. soil contamination, prevalence in dogs and cats, and seroprevalence in humans to inform control strategies.

## 2 Materials and methods

### 2.1 General model description

In this study we model the burden of toxocariasis by combining population-level seroprevalence data from Ma et al. (2020) with detailed clinical case data from Halsby et al. (2016) to provide estimates for the number of cases of the three principal symptomatic manifestations of toxocariasis: common toxocariasis, OLM, and VLM for the 28 countries for which data are available (Ma et al., 2020).

For the purposes of this study, we define common toxocariasis as a non-specific febrile illness in which various combinations of the following are present: abdominal pain, anorexia, nausea, vomiting, headaches, lethargy, and behavioural or sleep disturbances. In many cases covert and common toxocariasis are used interchangeably to describe the spectrum of *Toxocara* disease that does not require medical attention. However, for the purposes of this study we define both covert and common toxocariasis as sub-clinical, in the sense that neither manifestation presents as serious enough as to require specialised medical attention beyond treatment of symptoms at the most. Covert toxocariasis is herein defined as the asymptomatic form of toxocariasis, thus, common toxocariasis represents the mild symptomatic manifestation of the disease. VLM is defined as a severe systemic infection characterised by hepatic (hepatomegaly, hepatic granuloma), and pulmonary (coughing, wheezing, shortness of breath) symptoms, although there is some symptomatic overlap between common toxocariasis and VLM. Toxocariasis disease manifestations in general exist on a spectrum of severity (Auer and Walochnik, 2020) - for the purposes of this study we have chosen to define VLM as the most severe form of (non-ocular) toxocariasis, whereas milder forms of the disease are grouped under common toxocariasis. In this study, we have further defined asymptomatic cases as covert. OLM is defined as migration of *Toxocara* spp. larvae into the eye, leading to unilateral and progressive degradation of sight over days-weeks leading to the potential for permanent monocular vision loss (McGuinness and Leder, 2014). Finally, in this study, we have not explicitly considered clinical NT, as there have only been approximately 20 cases reported between 1950 and 2000 (Auer and Walochnik, 2020), but capture subclinical consequences of neurological larval migration through effects on cognition in children, for which see below.

To carry out the DALY calculations, we initially predicted the total number of seropositive individuals within the population based on the seroprevalence data from selected countries (Ma et al., 2020), for which co-located data on animal infections were also available. We then extrapolated the estimated number of VLM and OLM cases per age group based on the UK data between 2000 and 2009 (Halsby et al., 2016), and then divided this value by 10 to yield an average per year as the data used herein covers a 10-year period, assuming that this would be consistent across populations, although the demographic proportions would naturally vary. We then subtracted the number of expected clinical cases from the total seropositive population to yield the expected number of exposed, subclinical, and common *Toxocara* spp. infections within the population per year (see Section 2.3.). For clinical cases Halsby et al. (2016) reported that 21.7% of cases had ocular involvement, and therefore, we have assumed that the remaining 78.3% of cases are VLM. We further assumed (see Section 2.3.) that 30% of sub-clinical cases per year will be symptomatic, to yield our final estimate for the number of common cases within each country. It must be stated here, however, that this is an assumption based on the statement from the CDC that the majority of toxocariasis cases are not symptomatic (CDC, 2024). We therefore assume that < 50% of cases will display symptoms. The figure of 30% was thus chosen as a conservative “best guess” estimate which balances the potential to over- or under-estimate the burden of this disease manifestation. These general principles are outlined in the equations below:

• Seropositive individuals = Total population × Seroprevalence
• Estimated total cases (per age group) = Seropositive individuals × Percentage cases (per age group)
• Estimated severe cases (per year, per age group) = Estimated total cases (per age group) × Estimated percentage symptomatic cases
• Estimated common cases (per age group) = Estimated total cases (per age group) – Estimated severe cases (per age group)
• Estimated visceral cases (per age group) = Estimated severe cases (per age group) × 0.783
• Estimated ocular cases (per age group) = Estimated severe cases (per age group) × 0.217
• Estimated symptomatic common cases (per age group) = [Estimated seropositive individuals (per age group) – Estimated severe cases (per age group)] × 0.3

For a full breakdown of all calculations for all countries see Supplementary Table S1. To calculate 95% confidence intervals for DALY estimates herein we used the lower and upper bounds of the 95% confidence intervals for seroprevalence estimates provided by Ma et al. (2020). For a full breakdown of all model input parameters see Table 1.

**Table 1:** Model input parameters.

Parameter	Value	Source
Disability weight OLM	0.017	GBD (2019)
Disability weight VLM	0.011	Noguera Zayas et al. (2021); GBD (2019)
Disability weight common toxocariasis	0.006	GBD (2019)
Disability weight cognitive impairment in children	0.031	GBD (2019)
Duration OLM	1	Present study
Duration VLM	0.278	Noguera Zayas et al. (2021)
Duration common toxocariasis	0.15	Present study
Duration school missed	0.15	Present study
Proportion seropositive individuals symptomatic for common toxocariasis	0.3	Present study
Estimated proportion seropositive individuals symptomatic for VLM/OLM	Varies by age group (see Supplementary Table S1)	Halsby et al. (2016)
GDP per capita (USD)	Varies by country (see Supplementary Table S1)	World Bank (2022)
Cost of GP visit (USD)	45	Brook Medical Centre (2013)
Cost of sight loss per year (UK) (USD)	8672	Pezzullo et al. (2018)
Cost of laboratory testing (UK) (USD)	58	Assumed
Cost of anthelmintic treatment (UK) (USD)	12	UK (2023)
Percentage GDP healthcare spending	Varies by country (see Supplementary Table S3)	World Bank (2022)
Correction factor for GDP per capita	GDP per capita (Country)/GDP per capita (UK), 2022	
Correction factor for healthcare as percentage of GDP	Healthcare spend (Country)/Healthcare spend (UK), 2022	

**Fig. 1:**
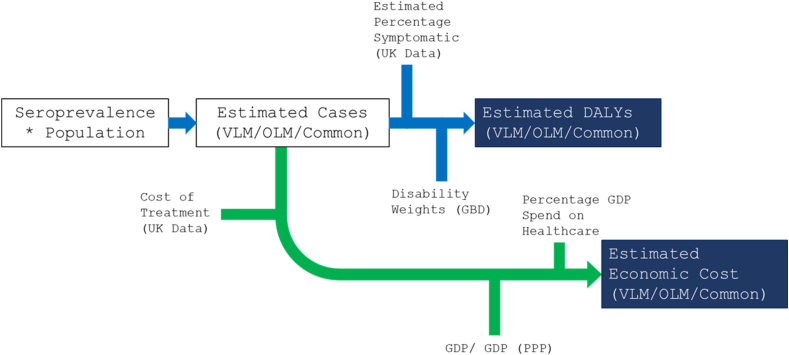
Summary of modelling approach. Initially, the number of seropositive individuals was estimated within each population, from which the estimated number of cases for VLM, OLM, and common toxocariasis were estimated. Finally, this allows for the estimation of DALYs, and economic impact based on a variety of input parameters. Blue lines indicate disease-related inputs. Green lines represent economic related inputs. Dark blue boxes represent final model outputs. *Abbreviations*: VLM, visceral *larva migrans*; OLM, ocular *larva migrans*; DALY, disability-adjusted life-year; GBD, global burden of disease (study); GDP, gross domestic product; PPP, purchasing power parity.

To estimate the economic impact of the different disease manifestations of toxocariasis, we followed the same approach as for the estimation of the number of cases, extrapolating based on UK data. This was carried out to maintain consistency across the analysis, and account for significant discrepancies in the availability of data for specific countries. Correction factors were applied to economic estimates based on the gross domestic product (GDP) per capita differences between the UK and the country in question, and the GDP percentage healthcare spend (Fig. 1).

### 2.2 DALY calculation

The following modified prevalence-based DALY formula has been used in this study, based on years lost to disability (YLD) (adapted from WHO, 2020):

DALY(c,s,a,t) = YLD(c,s,a,t) for given cause c, age a, sex s and year t,

where:

YLD(c,s,a,t) = I(c,s,a,t) × DW(c,s,a) × L(c,s,a,t),

where:

I(c,s,a,t) = number of incident cases for cause c, age a and sex s,
DW(c,s,a) = disability weight for cause c, age a and sex s,
L(c,s,a,t) = average duration of the case until remission in the reference year.

As toxocariasis did not lead to any reported deaths during the reporting period of the example data used (Halsby et al., 2016), further discussed below, we have only considered years lost to disability and not years of life lost (YLL).

Finally, we made use of a prevalence-based DALY calculation where the person-time lived in the specified health state for the reference year only is considered. Therefore, the average duration of the case is only considered for disease manifestations that last for less than one year.

Final estimates of DALYs per country per year, globally, and per 100k people were obtained by the sum of all DALYs per country for each of the disease manifestations and cognitive impairment in children.

### 2.3 Model assumptions

In order to address the issue of the likely underestimation of toxocariasis, we made use of two studies to parameterise and ground our modelling approach. The first is a study making use of detailed longitudinal data from the UK on all cases of laboratory-confirmed toxocariasis in England and Wales between 2000 and 2009. This data was collected from two sources: the clinical records from the Department of Clinical Parasitology at the Hospital of Tropical Diseases (London) (HTD), and the Lab Base (LB) surveillance system, a routine laboratory reporting system used by the National Health Service (NHS) in operation since 1975. The data used as the foundation for the current study is from 2000 to 2009 (Halsby et al., 2016), and is broken down by age group (10-year intervals). This data provides information on ocular involvement (Halsby et al., 2016), allowing for an estimation of the proportion of clinical cases manifesting as OLM, wherein the authors report that, where information was available, 21.7% of cases had ocular involvement (Halsby et al., 2016). We have therefore, assumed that the remaining 78.3% of clinical cases are due to VLM. As Halsby et al. (2016) do not provide a breakdown of cases by sex per age group, and as sex was not provided for 21 cases in the HTD database, we have assumed a 50:50 split of sex within the population, which roughly corresponds to the sex ratio of the England and Wales population between 2000 and 2009 (49% male:51% female) World Bank (2022). Further to this, for the life expectancy calculation we have taken the mean of the life expectancy of males and females between 2005 and 2007 which was 77.5 for males, and 81.7 for females CENSUS (2013). This leads to a mean life expectancy of 79.6 years. For countries other than England and Wales, including the estimation provided for the whole of the UK at 2020 population, we have taken the life expectancy in 2020 provided by datacommons.org. As the data is taken over a 10-year period, DALY calculations were carried out initially on the data from the full 10 years, after which the final value was divided by 10 to give an estimation per year. This study represents one of the most detailed breakdowns of clinical cases of toxocariasis. Although studies of comparable detail are available, for example in Poland (Borecka and Kłapeć, 2015), Halsby et al. (2016) was chosen as the clinical data is broken down by severe disease manifestation and age group, allowing for estimations across the whole population to be made. The second study is a systematic review of seroprevalence data from a selection of 28 countries (Ma et al., 2020). This study provides average estimated seroprevalence for humans from a wide range of studies, and from countries broadly representative of their respective geographical regions and continents, in addition to data on soil contamination, and prevalence of *Toxocara* eggs in the faeces of dogs and cats. Based on this study we have assumed that seroprevalence serves as an approximate measure of the total number of potential infections per year (including all disease manifestations). This assumption is based on antibody persistence levels, with antibodies being shown to decline between 3 months and 2 years post-exposure (Żarnowska et al., 2008; Wiśniewska-Ligier et al., 2012). Most major countries are represented, although unfortunately there is relatively little data available for sub-Saharan African countries, with only Nigeria and Kenya represented.

#### 2.3.1 Common and covert toxocariasis

For the purposes of defining asymptomatic, clinical, and common symptomatic cases, we define a clinical case as any case exhibiting symptoms severe enough to warrant specialist medical attention necessitating laboratory confirmation. A common case is thus, defined as any case exhibiting symptoms mild enough that no specialist medical attention was sought, and was self-limiting. However, here, we wish to clarify that these symptoms will exist on a broad spectrum (Taylor et al., 1988), potentially overlapping with those cases severe enough to warrant laboratory confirmation. For the purposes of this analysis, however, this will include mild but symptomatic manifestations of the condition. For those cases in which the patient does not exhibit any symptoms, termed herein as covert toxocariasis, a DW of 0 has been assigned. For those cases which exhibit mild symptoms a DW of 0.006 (0.002–0.012) has been assigned, and which herein are referred to as common toxocariasis. The latter DW corresponds to “infectious disease, acute episode, mild” which covers a range of different infections (GBD, 2019). This DW has been chosen to represent as “average” for the wide spectrum of possible symptoms for the sub-clinical manifestation of the disease, and is meant as an approximation of this range. Accurate information on the occurrence of common toxocariasis is not available (Halsby et al., 2016). According to the CDC “Many people infected with *Toxocara* do not have symptoms” (CDC, 2024), thus, we have assumed that 30% of seropositive individuals not exhibiting either OLM or laboratory-confirmed VLM will suffer from common toxocariasis, and the remaining 70% covert toxocariasis, exhibiting a mild and self-limiting febrile illness. However, it must be unequivocally stated that this proportion represents an arbitrarily chosen value thought to best balance the risk of over- or under-representing the potential impact of the disease. Nevertheless, in the absence of detailed data, this must be taken as indicative of the potential impact and is not based on clinical knowledge as this is not available. Finally, we have assumed a duration of 8 weeks, as cases typically resolve within 4–12 weeks (CDC, 2024), therefore we have chosen the midpoint of this range to represent an average expected duration of mild symptoms.

In school-age children, we have also undertaken to estimate DALY for short term impaired cognitive function due to disease symptoms (Walsh and Haseeb, 2012). For this indication, the following DW has been used: 0.031 (0.018–0.048) which is the DW for mild intellectual disability (GBD, 2019). Although impact on childhood education and development has been hard to quantify (Gottfried, 2010), this DW is meant to closely approximate a time-limited reduction in learning capacity due to ill-health. The duration has been assumed to be the same as the duration for a symptomatic common toxocariasis case, i.e. 8 weeks. This has been chosen to represent not only the time lost directly for being absent from school due to illness, but also decreased energy during recovery, and the need following absence to “catch up” with lost work. Therefore, this should be taken as an approximation of the full effects of the episode of illness on the child's life, in addition to the symptomatic effects of the disease. We did not take into account the potential direct effects of toxocariasis on cognitive development, which could theoretically have long-term health and economic implications, as such impacts are still under debate (Walsh and Haseeb, 2012).

#### 2.3.2 Visceral larva migrans

As severe cases are rare, we have followed the same assumptions used by Noguera Zayas et al. (2021), namely, a DW which corresponds to “mild abdominopelvic problems due to ascariasis/hookworm”: 0.011 (0.005–0.021) (GBD, 2019). However, this again only represents an approximation of the wide range of symptoms that can affect a patient suffering from VLM, and is intended to be an indicative “average” to preliminarily estimate potential burden. Similarly, in relation to the duration of the disease we have followed Noguera Zayas et al. (2021) and have assigned a duration of 14.5 weeks (0.278 years). As 78.3% of cases in the HTD reported no ocular involvement, we have assumed that this corresponds to the proportion of clinical cases with VLM. This assumption is based on the data used for these calculations being derived from laboratory-confirmed cases. Laboratory confirmation, according to the authors, is unlikely to have been ordered for a mild symptomatic case, and thus the authors also stated that likely the true prevalence of toxocariasis is underestimated (Halsby et al., 2016).

#### 2.3.3 Ocular larva migrans

According to the percentage of cases with ocular involvement in HTD, we have assumed that 21.7% of all clinical cases have ocular involvement and a monocular permanent vision loss, with a DW of 0.017 (0.009–0.029) (“monocular distance vision loss due to meningitis” (GBD, 2019).

### 2.4 Economic burden calculation

To estimate the cost of each disease manifestation the total estimated number of cases was multiplied by the total cost of treatment interventions, with a correction factor applied to take account of differences in GDP per capita and GDP spend on healthcare. In the case of calculating the cost of missed work, the duration of the disease manifestation was multiplied by 0.3. Hence, we have assumed the individual would be unavailable for work for an average of 30% of the total disease duration. This is to take account of the self-limiting nature of common toxocariasis, and the fact that most individuals will not have access to long term sick-pay at their full salary cost. However, again, it must be categorically stated that this is an arbitrarily assigned value to represent a balance between under- and over-representing the impact of toxocariasis and is not based on any clinical data. We then multiply by the GDP per capita of the country in question (see Supplementary Table S1 for full breakdown of calculations). Purchasing power parity (PPP) is an economic measure of the difference between the price of a set of specific goods used to compare the purchasing power of countries’ currencies (OECD, 2024). To take account of this, for all economic calculations we have also adjusted for PPP (see Supplementary Table S3).

For the cost of a general practitioner (GP) visit we have assumed a cost of $45 (USD) (£36 (GBP)) based on information supplied by the Brook Medical Centre (2024), a UK (Derby, England) based GP surgery. All currency conversions are based on the exchange rates at the time the analysis was conducted – Q4 2023. Although actual costs will vary by country and region, this has been chosen as an average approximation, based on the location of the surgery in a medium-sized urban area within the UK. For the cost of sight loss per year we have estimated an annual cost per person (which includes all costs of medical intervention and loss of income etc) from Pezzullo et al. (2018) as follows: sight loss affects 1.93 million individuals within the UK, with costs estimated at £7.2–19.5 billion across the whole of the UK; therefore, we have taken the simple arithmetic mean [(7.2 + 19.5)/2], and divided by the total population of the UK, giving a final figure of £6917 per person per year ($8672). For the cost of laboratory testing, we have taken the UK National Health Service (NHS) laboratory cost of a *Cryptosporidium*/*Giardia* ELISA (£46; $58), assumed based on authorsʼ experience. However, again, this represents an estimation and an average cost of testing for a range of cases. We also do acknowledge that for many laboratory-confirmed cases, the patient will likely have been tested for multiple other infections prior to testing for *Toxocara*, as toxocariasis is rarely included in differential diagnoses (Auer and Walochnik, 2020). Finally, for the cost of symptomatic treatment, we have assumed the NHS standard charge of £9.65 ($12) for a prescription. This cost approximates the average cost of generic prescription medication in the UK. For countries other than the UK, correction factors are applied to the sum of all economic costs calculated from these parameters. It must, nonetheless, be stated that these costs will only serve as an approximation for estimation and can deviate from the true values in different countries. However, because for many of the countries considered, healthcare data are not freely or easily accessible the purpose of the study is to give an initial rough estimate of the potential costs.

### 2.5 Sensitivity analysis

**Fig. 2:**
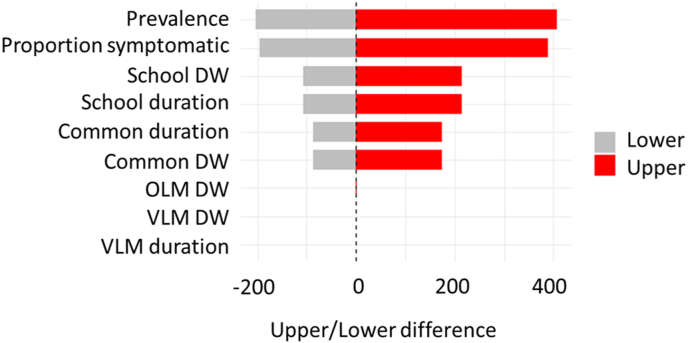
Sensitivity analysis for model input parameters showing the difference between the upper and lower values obtained by altering input parameters by ± 20%. The dotted line indicates the mean value used for the reported outputs.

Sensitivity analysis was carried out to determine which parameters had the greatest effect on model outputs. Each input parameter was varied by ± 20%. We found that seroprevalence had the greatest effect on output value, followed by the assumed percentage of symptomatic cases, the DW and duration for missed school, common toxocariasis duration, and DW. Parameters chosen for OLM and VLM had little overall effect on model outputs (Fig. 2).

We then carried out further sensitivity analysis on the percentage of common cases which are expected to be symptomatic. Initially, we examined the effect on DALYs by a reduction from 30% to 15% of the percentage of common cases expected to be symptomatic, and doubling the number of cases of VLM. A reduction to 15% in the number of common cases expected to be symptomatic had a large effect on the number of DALYs expected to be lost, leading to a ∼49% reduction in estimated DALYs (489.83 *vs* 976.52 including cognitive impairment). Doubling the number of cases of VLM had a negligible effect (< 0.001% difference; 976.52 *vs* 976.57 total DALYs including cognitive impairment).

## 3 Results

### 3.1 DALY estimates

In the UK, for common toxocariasis we estimated 434.54 (95% confidence interval, 95% CI: 380.22–470.75) DALYs per year, and for the potential impact of temporary cognitive impairment in school-age children (< 18) we estimated 538.83 (95% CI: 471.476–583.732) DALYs. The estimated DALYs for VLM and OLM were 0.047 (95% CI: 0.041–0.051) and 3.102 (95% CI: 2.714–3.361), respectively. We estimated a total of 976.519 (95% CI: 854.454–1057.895) DALYs attributable to toxocariasis. This translates to a DALY per 100k of 1.456 (1.274–1.578). As such, VLM and OLM accounted for < 0.5% of the total.

In the other countries, we found the highest estimated total DALYs in India with 17,155.433 (95% CI: 8577.716–27,448.692); China with 12,945.691 (95% CI: 10,356.553–15,534.829); Nigeria with 5820.158 (95% CI: 2513.25–9523.895); and Brazil with 3649.035 (95% CI: 2736.776–4561.294) (Supplementary Table S2). Of the high-income countries examined, the USA (1842.809 [1228.539–2661.835], the UK (976.519 [854.454–1057.895]), and Argentina (964.11 [716.196–1239.57]) had the highest overall DALYs due to toxocariasis (see Fig. 2).

**Fig. 3:**
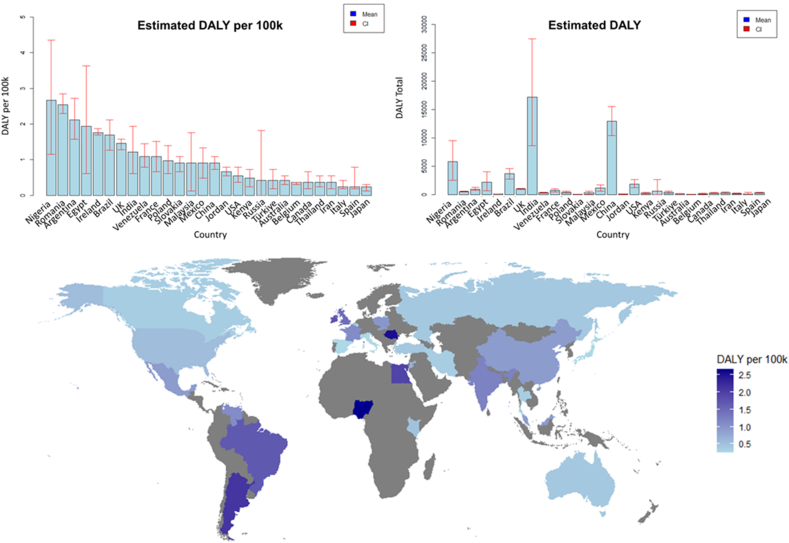
Summary of estimated DALYs per 100k, estimated DALYs, and world map showing the estimated DALY per 100k per country for which data was available. Countries are shaded based on the DALY per 100k, with darker indicating a higher number; those with no data are shaded grey. A full summary of all country seroprevalences and DALYs can be found in Supplementary Table S1. *Abbreviation*: CI, 95% confidence interval.

When considering DALYs per 100k population (Fig. 3), Nigeria (2.663 [95% CI: 1.15–4.358]), Romania (2.542 [95% CI: 2.3–2.845]); Argentina (2.118 [95% CI: 1.574–2.724]); and Egypt (1.937 [95% CI: 0.605–3.632]) returned the highest values. Ireland (1.755 [95% CI: 1.695–1.876]), and the UK (1.456 [95% CI: 1.274–1.578]) were also estimated to have relatively high DALYs per 100k compared to other high-income countries. When accounting for population, India (1.211 [95% CI: 0.605–1.937]), and China (0.908 [95% CI: 0.726–1.089]) fell within the mid-range of DALYs per 100k. High-income countries in both cases account for 30–40% of countries in the top 10.

The total DALYs, based on the 28 included countries were 51,751.43 (95% CI: 30,992.39–77,053.33), of which 23,084.00 (95% CI: 13,824.06–34,370.56) due to common toxocariasis, and 28,624.17 (95% CI: 17,141.83–42,619.50) due to temporary cognitive impairment (Supplementary Table S2). Severe clinical manifestations VLM and OLM accounted for a negligible proportion (< 50 DALYs) of the total. Finally, using the global average seroprevalence of 19% (95% CI: 17–21%) (Ma et al., 2020), we estimated that, across all countries globally, 91,714 (95% CI: 82,060–101369) DALYs are lost due to toxocariasis, of which 40,912 (95% CI: 36,605–45,218) are attributable to common toxocariasis, and 50,731 (95% CI: 45,391–56,071) to cognitive impairment in children.

### 3.2 Correlation of prevalence in companion animals, soil contamination, and prevalence in humans

**Fig. 4:**
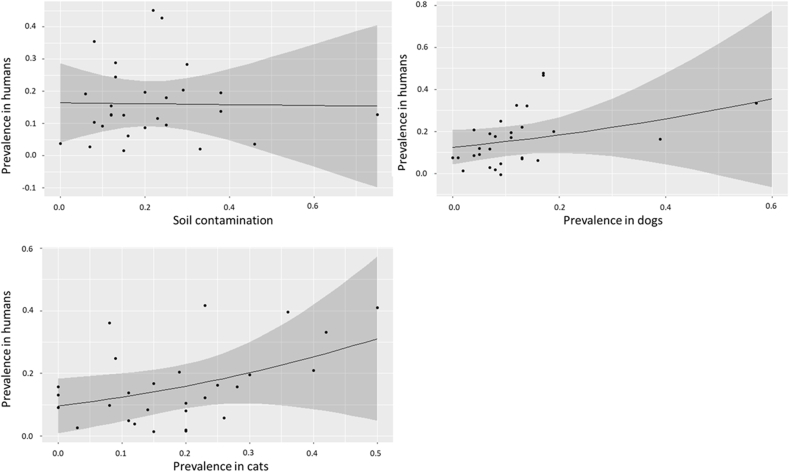
General linear models of correlation coefficients for the seroprevalence of *Toxocara* spp. in humans *vs* the contamination of soil with *Toxocara* spp. eggs, prevalence in dogs, and prevalence in cats. Data source: Ma et al. (2020).

We then sought to examine the correlation between prevalence of *Toxocara* in dogs and cats, soil contamination, and prevalence of *Toxocara* antibodies in humans based on the data available for the 28 countries in Ma et al. (2020). There was no detectable correlation between prevalence in humans and soil contamination (*R* = −0.02, *P* = 0.93) or prevalence in cats or dogs and soil contamination (*R* = −0.03, *P* = 0.86). There were low and (marginally) significant positive correlations between the prevalence in dogs and humans (*R* = 0.36, *P* = 0.06), and cats and humans (*R* = 0.45; *P* = 0.02) (Fig. 4).

### 3.3 Economic estimate

The total annual economic impact of VLM, and OLM in the UK was estimated at $60,239 and $367,553 respectively. Based on data from Halsby et al. (2016) we estimate a total of 15 cases of clinically confirmed VLM and 4 of OLM for the whole UK. Per case costs were estimated as $4016 for VLM and $91,888 for OLM. The total economic impact of common toxocariasis was estimated at $194,257,373. When adjusted for PPP this value was corrected to $231,847,416. The total cost of toxocariasis in the UK was estimated to be 0.006% of the total GDP and the annual per capita economic impact $3.48. For a full breakdown of all economic estimates see Supplementary Table S3.

**Fig. 5:**
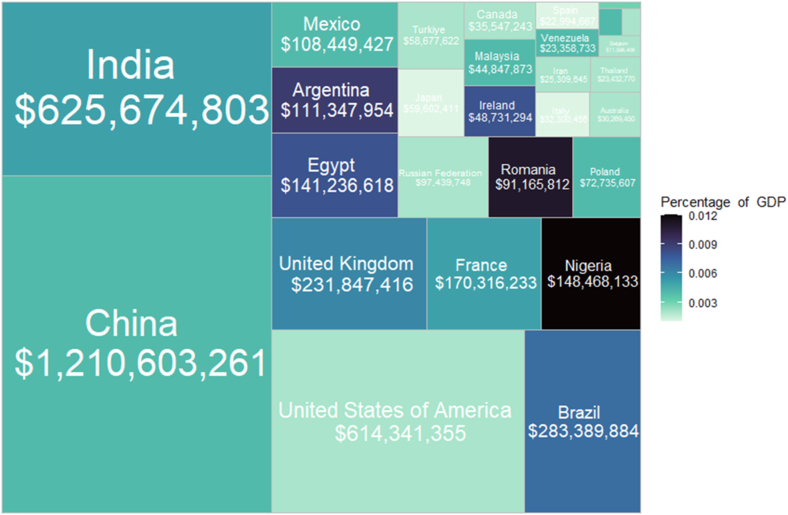
Waffle chart summary of estimated economic impact adjusted for purchasing power parity (PPP). The area of squares corresponds to estimated unadjusted economic cost, with shading corresponding to that figure as a proportion of gross domestic product (GDP) per country for which data was available.

For the other countries, we estimated that China ($717,051,705), the USA ($614,341,355), the UK ($194,685,166), and India ($178,358,873) experienced the highest unadjusted economic impact due toxocariasis. When adjusted for PPP the highest economic impact was in China ($1,210,603,261), India ($625,674,803), the USA ($614,341,355), and Brazil ($283,389,884) (Fig. 5). However, these metrics skew to larger countries and economies. When expressed as a proportion of the GDP, the impacts were greatest in Nigeria (0.012%), Romania (0.011%), Argentina (0.009%), and Egypt (0.008%). The greatest PPP adjusted per capita impacts were in Ireland ($9.70), Romania ($4.64), the UK ($3.46), and France ($2.64).

## 4 Discussion

To the best of our knowledge, this study represents the first quantitative impact estimation of human toxocariasis. We have developed a structured approach and transparent assumptions to assess the burden of this poorly known disease, provided for the first time a rough indication of the economic costs and explored the correlation between prevalence data in human and animal reservoirs. Our preliminary findings show that toxocariasis may exert a significant burden on global health and national economies. Because companion and feral dogs and cats and their environment are the main reservoirs for human infection, this burden can in theory be reduced *via* straightforward control measures targeting these reservoirs, complemented by hygiene to reduce ingestion of parasite eggs from contaminated environments (Overgaauw and Van Knapen, 2013). Our findings should serve as a call to action to further clarify the true burden of toxocariasis globally, and highlight the importance of its inclusion within future GBD studies.

Herein we report a number of novel findings related to the burden of toxocariasis. First, previously DALYs for toxocariasis were estimated as part of larger studies considering multiple diseases as is the case for the systematic review and estimation of zoonosis DALYs (zDALYs) in Paraguay carried out by Noguera Zayas et al. (2021). Due to the nature of this study, the authors only considered clinical cases of VLM, and did not consider OLM or common toxocariasis. However, these severe clinical manifestations are relatively rare, and do not account for the full disease burden of toxocariasis. Indeed, by our own estimations, they account for less than 0.5% of DALYs attributable to toxocariasis globally. This is further indicated by the difference between seroprevalence estimates globally (Ma et al., 2020) and recorded cases of clinical toxocariasis at the national level (Halsby et al., 2016; Noguera Zayas et al., 2021), indicating variation in the rate of detection, confirmation, and reporting of clinical cases. Following on from this, we used global seroprevalence estimates as a proxy to estimate the potential burden of toxocariasis. This was done as, although the clinical significance of a positive serological test for *Toxocara* antibodies has yet to be firmly established, there is an urgent need to better understand its potential burden to inform public health policies. Thus, in the absence of direct clinical information for common toxocariasis, which is by definition sub-clinical and self-limiting, the use of seroprevalence estimates represents the best available option to assess the potential burden of common toxocariasis at the time of writing.

To estimate the DALYs lost due to common toxocariasis, we made use of a detailed global systematic review and meta-analysis (Ma et al., 2020), which draws on data from previous systematic reviews covering over 265,327 individuals across 71 countries (Rostami et al., 2019), combined with systematic review and meta-analysis of prevalence of *T. canis* in dogs (Rostami et al., 2020a); *T. cati* in cats (Rostami et al., 2020b); and soil contamination (Fakhri et al., 2018). Using these data, in the 28 countries for which prevalence data was available, we estimated the total DALYs due to common toxocariasis to be 23,084. Using the global average seroprevalence (19% [95% CI: 17–21%]) across all countries (Ma et al., 2020), we estimated a global burden of 40,912 (95% CI: 36,605–45,218) for common toxocariasis. It has been suggested that the burden of toxocariasis has been underestimated (Halsby et al., 2016; Hotez, 2020). Our estimates appear to be in line with this. When compared to the estimated burden of food-borne zoonoses (estimated for the food-borne transmission route only), toxocariasis exerts a similar burden to that of *Echinococcus granulosus* (39,950 DALYs), *Giardia* spp. (26,270 DALYs), and Shiga toxin producing *E. coli* (12,953), and significantly more than *Trichinella* spp. (550 DALYs) (Havelaar et al., 2015). It has previously been suggested that the burden of toxocariasis could be similar to that of STHs (Hotez, 2020). Our estimates, however, are significantly below those of STHs, which typically exert burdens in the > 1 million DALY range (Pullan et al., 2014; Montresor et al., 2020). Nonetheless, this does come with an important caveat. Our estimates are based on a relatively small number of countries, and a single global average estimate, from a set of studies considered as part of a systematic review into global seroprevalence estimates (Ma et al., 2020). Furthermore, this dataset had available data from only three African countries (Nigeria, Egypt, and Kenya), representing only around a quarter to a third of the population of Africa. In the interests of consistency in our analysis, we did not make use of data from any other African countries from other studies, thus, the majority of Africa has not been considered in this analysis. In Asia, although data was available from India, Thailand, Malaysia, and China, we did not have data available for Indonesia, Pakistan, Bangladesh, and Myanmar, which collectively account for more than 500 million inhabitants. This represents a significant gap in the data, and one which should be explored in future studies.

A further novel finding of this study builds on previous work which has suggested that infection with toxocariasis can lead to cognitive impairment in both children (Walsh and Haseeb, 2012) and adults (Song et al., 2023). This merited further exploration, and constitutes a potential hidden burden of toxocariasis not considered previously. While we did not consider the direct or long-term impacts of *Toxocara* spp. infection on learning ability or consequent life prospects, which could be considerable but highly uncertain, we did estimate the temporary effect of common toxocariasis on the interruption of learning. Indeed, this approach is unique to DALY calculations in general, and to the best of our knowledge has not been considered to date by similar studies assessing the burden of zoonoses and STHs (Pullan et al., 2014; Havelaar et al., 2015). We found that the potential burden of toxocariasis due to short-term cognitive impairment was greater than that even for common toxocariasis, with up to 28,624 DALYs lost in the 28 countries for which seroprevalence data was available. This was in spite of a very moderate effect size based on short term self-limiting disease. Based on the global average seroprevalence we estimated 50,731 (95% CI: 45,391–56,071) DALYs lost due to cognitive impairment in this way in children. Based on our analysis, this accounts for the single largest source of DALYs lost due to toxocariasis. This is particularly relevant as many socioeconomically disadvantaged children are disproportionally affected by toxocariasis (Hotez and Wilkins, 2009; Smith et al., 2009; McGuinness and Leder, 2014). This finding further suggests that it may be of benefit to consider effects of infection on school learning in future studies on STHs, and other parasitic diseases which also disproportionately affect children in socioeconomically disadvantaged areas.

An issue that has hampered attempts to better understand and quantify the burden of toxocariasis has been the lack of recognition of the disease, for example, within GBD studies, and, unlike the STHs (Pullan et al., 2014), no toxocariasis DWs are available (Noguera Zayas et al., 2021). Thus, we were required to make approximations based on the closest proxy to the symptoms of each manifestation. For VLM, we followed Noguera Zayas et al. (2021) in assigning a DW corresponding to abdominopelvic ascariasis symptoms. However, this likely does not capture the true picture of VLM, as symptoms are wide-ranging and can be severe (Despommier, 2003). For OLM, we simply made use of the DW for monocular vision loss, and this does not account for other corollary symptoms which may be experienced. Similarly, for common toxocariasis, we made use of a DW corresponding to an acute episode of mild infectious disease, as the closest approximation of a mild and self-limiting condition. We nonetheless recognise that this may underestimate the burden, and future studies should focus on developing specific DW for common toxocariasis as well as specific manifestations of VLM. Difficulties in disaggregating symptoms of toxocariasis and avoiding double counting have held back previous attempts to quantify the health and economic burdens of toxocariasis (Torgerson and Budke, 2006), and are common to parasitic infections that often involve co-morbidities (Payne et al., 2009). DALYs are now so well established as a metric and widely used to guide health policy and justify research efforts, yet there are still to date no DWs assigned for toxocariasis; however, to put off estimates for toxocariasis due to methodological difficulties carries a serious risk of perpetuating the neglect of this parasite by governments and communities worldwide and allowing high levels of continuing preventable illness. Consequently, we address this gap in a way that allows uncertainties to be reduced with further methodological refinement and data collection. Finally, with regard to our DALY calculations, in this study we did not consider the most severe manifestation of toxocariasis, NT. This was for a variety of reasons. Mainly, although NT is the most severe manifestation, it is also by far the rarest, with only 20 cases reported between 1950 and 2000 (Auer and Walochnik, 2020), with another study stating that a total of 99 cases were reported worldwide as of 2018 (Chen et al., 2018). No cases of NT were reported in the study by Halsby et al. (2016), which formed one of the bases for the parameterisation of our model. However, this is an aspect of toxocariasis that should be considered in future work, as although NT is rare, it is severe, often long-lasting, difficult to diagnose and treat, and can, in rare cases, lead to deaths. Nevertheless, it was judged to be beyond the scope of the current study. Cognitive impairment might, however, be considered a less severe consequence of larval migration in neurological tissue and was found to have a large health impact.

We also examined the correlation between the prevalence of *Toxocara* in companion animals, humans, and soil contamination. We found a moderate positive correlation between prevalence in cats and dogs and seroprevalence in humans, although this correlation was statistically significant in cats only. This finding is of interest as traditionally *T. canis* has been thought to be the primary driver of human infection, with *T. cati* only considered peripherally (Maciag et al., 2022), although it is long been considered likely that the potential role of *T. cati* infection in zoonotic toxocariasis is underestimated (Fisher, 2003; Maciag et al., 2022). However, until such a time as *Toxocara* serological testing can differentiate between antibodies against *T. canis* and *T. cati* (Poulsen et al., 2015), this will likely remain an open question. The correlation between prevalence in cats and humans may also be due to the closer proximity of humans to cats, particularly in high-income countries where there is a relative paucity of stray cats and dogs, and with cats generally kept within the home and using a litter box to defecate, which can also lead to household contamination (Tateo et al., 2023). With regards to findings in dogs, it must also be stated that although the dataset made use of data from over 12 million dogs worldwide, a relatively small proportion (< 100,000) of the animals sampled across all studies were stray dogs (Rostami et al., 2020a). Given the close association between stray dogs and the spread of zoonoses (Otranto et al., 2017), this is a further aspect that requires attention in future work. These findings also indicate that more effective control measures in companion animals could help reduce the incidence of toxocariasis in humans, although further work is necessary to clarify this, especially given that prevalence of egg shedding in dogs in Europe does not appear to have declined in recent decades (Overgaauw and Nijsse, 2020). Perhaps surprisingly, we did not find any correlation between soil contamination and seroprevalence in humans. However, this must be taken with the caveat that soil contamination data are scarce and localised, while we considered the seroprevalence of the whole population, rather than corresponding localities or the sub-sections of the population most likely to be affected by contaminated soil - young children. Indeed, this would be an important area to explore in future studies, as significant green space contamination with *Toxocara* eggs has been reported in, for example, the UK (Airs et al., 2023), highlighting the need for more focused country-specific studies, and greater methodological standardisation of soil studies so that results are comparable. It would be of interest in future studies to examine the relationship between soil contamination and seroprevalence in children at local community level, as well as change in both factors over time following greater control efforts.

Finally, we sought to provide a preliminary estimate of the potential economic impact of toxocariasis infection in the countries for which data was available (Ma et al., 2020). We estimate the annual cost of toxocariasis to be $2.5 billion ($4.5 billion when adjusted for purchasing power parity) in the 28 countries considered herein. The overwhelming majority of this estimated cost is due to common toxocariasis, once again highlighting the importance of considering this disease manifestation in future work. However, again this represents only the tip of the iceberg and there is good reason to believe that our estimates fall on the lower end of the plausible range of impact.

This is the case as firstly, (common) toxocariasis has been widely acknowledged to be significantly underreported (Halsby et al., 2016; Hotez, 2020). This is related to the fact that (i) toxocariasis is a self-limiting disease that does not cause severe enough symptoms in the vast majority of cases for medical attention to be sought, and (ii) by the difficulty in diagnosing toxocariasis, as unlike many other diseases, despite the high rates of seroprevalence reported, at least in the UK, testing for *Toxocara* spp. is not included in routine parasitology diagnostic panels. However, it must be reiterated that our assumption that 30% of cases will be symptomatic, of which only a very small number will be clinically confirmed, represents at this stage an arbitrary value chosen to represent a potential scenario, and this may indeed be an over- or under-representation of the true proportion. Based on sensitivity analysis (see Section 2.5.), this value has the second greatest effect after prevalence on the DALY values obtained in our analysis. This is further reflected by the fact that the overwhelming majority of DALYs are estimated to be lost due to common toxocariasis (including those lost due to temporary cognitive impairment in children), rather than the more severe disease manifestations. It is, therefore, imperative moving forward that there is increased clinical awareness of common toxocariasis, particularly at the primary care level where these cases present. In addition, it would be of great benefit to include *Toxocara* spp. in standard parasitological diagnostic panels, which would also begin to provide a wealth of data which could be used to estimate the true burden of this neglected disease. Furthermore, as toxocariasis has never been included in GBD studies, there are no bespoke DWs available for toxocariasis in any of its disease manifestations. In future GBD iterations, it may be of value to consider including specific DWs for toxocariasis. Finally, we made use of the data available in the format of the systematic review carried out by Ma et al. (2020), as this represents the most up-to-date and extensive review of toxocariasis seroprevalence available to date alongside data from animals and soil. However, there are significant gaps in the data, which future work should focus on filling and has begun to do so (Rostami et al., 2019). It would be of great value for future studies to carry out larger scale standardised seroprevalence sampling across a number of countries with large populations in understudied regions not represented in the data used herein, such as South Africa, the Democratic Republic of the Congo, Pakistan, and Indonesia. Finally, our data suggests a significant burden due to toxocariasis in a number of countries; however, this is based on a single seroprevalence estimate. Therefore, for those countries estimated to be most seriously affected by the disease, additional country-specific seroprevalence sampling would be of great value moving forward, coupled with a deeper analysis of country-specific databases for more severe disease manifestations. This would facilitate a clearer understanding of toxocariasis moving forward.

## 5 Conclusions

In this study, we sought to provide a preliminary estimate of the potential burden of toxocariasis globally. However, it must be stated that the figures provided here are preliminary estimates, and must be taken as indicative potential, and do not represent robust estimates of the true cost of toxocariasis. Nonetheless, we consider this to represent a necessary step forward to begin to understand the true impact of this disease. We estimate that toxocariasis, traditionally seen as a neglected zoonosis, may affect both high- and low/middle-income countries, with significant burdens estimated in countries such as Nigeria, India, Brazil, the UK, Romania, and the Republic of Ireland. We further estimated the effects of temporary cognitive impairment in children and common toxocariasis, aspects which have not to date been considered but could represent the greater part of the health impacts of this infection. Estimates for these effects are much higher than any previously reported DALY for toxocariasis and indicate that the true burden of the disease may be underestimated. Toxocariasis in humans is a poorly studied disease and thus our estimates are surrounded by large uncertainties. Nonetheless, our data point out that the true impact of toxocariasis may be caused by the undetected common cases, rather than the rare but serious clinical manifestations.

## Funding

This research did not receive any specific grant from funding agencies in the public, commercial, or not-for-profit sectors.

## Ethical approval

Not applicable.

## CRediT authorship contribution statement

**Alistair Antonopoulos:** Conceptualization, Methodology, Formal analysis, Visualization, Writing – original draft, Writing – review & editing. **Alessio Giannelli:** Conceptualization, Methodology, Writing – review & editing. **Eric R. Morgan:** Methodology, Writing – review & editing. **Johannes Charlier:** Conceptualization, Methodology, Project administration, Supervision, Writing – review & editing.

## Declaration of competing interests

Alessio Giannelli is employed at Inovet (Belgium). All other authors declare that they have no competing financial interests or personal relationships that could have appeared to influence the work reported in this paper.

## Data Availability

The data supporting the conclusions of this article are included within the article and its supplementary files.
